# 

**Published:** 1896-01-04

**Authors:** 


**Notices of Births, Deaths, and Marriages** are inserted in this column at a charge of 2s. 6d. for an announcement not exceeding four lines.

**Notice to Advertisers and Subscribers.**

All Advertisements, Orders for Copies of THE HOSPITAL and Business Communications should be addressed to **THE MANAGER, (Not to The Editor)**, THE HOSPITAL, 428, Strand, London, W.C.

The Cost of Subscription, post free, is as follows:—Per week, 2½d.; per quarter, 2s. 8d.; per half-year, 5s. 3d.; per annum, 10s. 6d. Foreign.—Per Annum, 15s. 2d.; payable, in all cases, in advance.

### NOTICE TO CORRESPONDENTS.

All MS., Letters, Books for Review, and other matters intended for the Editor should be addressed THE EDITOR, THE LODGE, PORCHESTER SQUARE, LONDON, W.

The Editor cannot undertake to return rejected MS., even when accompanied by stamped directed envelope.

**"Burdett's Hospital Annual," 1895,**

Sixth year of publication. 950 pp. Scarlet cloth, gilt lettered. Price 5s. A most complete guide to British, Colonial, Indian, and American Hospitals, Dispensaries, Nursing and Convalescent Institutions, and Asylums. A few complete sets of the preceding five issues of the "Annual" may still be obtained on application to the Publishers, THE SCIENTIFIC PRESS (Limited), 428, Strand, London, W.C.

**NOTICE.—Vol. XVIII. of "The Hospital" (April to September, 1895), in handsome cloth binding, is now on sale at the Office of this Journal. Price 7s. 6d. Cases for binding the Numbers contained in this Volume, 1s. 6d. Index to Vol. XVIII., 2½d. post free.**

**The Monthly Part for January, 1896, is now ready, price 6d., by post, 8d.**

### Scraps and Gleanings.

The ladies' sale of work for the National Orthopœdic Hospital held lately realised about £90.

The annual report of the Dublin Orthopœdic Hospital just issued is of a highly satisfactory character.

There have been no less than 1,010 cases of cholera during the two months of its outbreak in Egypt.

The kitchen concert, a time-honoured institution at the Edinburgh Royal Infirmary, was held recently.

In the December number of *Clinical Sketches* the Editor announces that the price will be reduced, commencing with the January number, from 1s. per copy to 6d.

It is gratifying to the authorities of the Royal Westminster Ophthalmic Hospital that one-tenth of last year's expenditure was defrayed by money contributed by grateful patients.

Mr. J. G. Bryant, late of the Great Northern and Metropolitan Hospitals, has been appointed special canvasser and collector to the Charing Cross Hospital from January 1st, 1896.

Two generous donors have forwarded £50 and £40 respectively to the funds of Freidenheim, a Home for the Dying, Upper Avenue Road, N.W., for which funds are greatly needed.

The Earl of Rosslyn has sent the Edinburgh Royal Infirmary and other institutions cheques amounting in all to £250, being the proceeds of his recent performances of "Diplomacy."

At the annual meeting of governors, &c., of the Tynemouth Jubilee Infirmary, North Shields, a resolution was unanimously carried to amalgamate the dispensary with the infirmary.

The sum still required to complete the extension work at Morley House Convalescent Home for Working Men, St. Margaret's Bay, Dover, is £4,000, and not £400, as erroneously stated.

A governor of the North-Eastern Hospital for Children has promised to give £25 towards the much-needed funds of the institution if nine other similar donations are forthcoming within a prescribed period.

A sum of about £320 has been received by the secretary of the East London Hospital for Children from Mr. F. W. Saunders, for the purpose of endowing one bed and two cots in the name of Miss A. M. Saunders.

The Derby Children's Hospital presents a satisfactory report on its workings during the past year. Some valuable internal improvements have been effected, and a new operation-room is in course of completion.

The Royal Albert Orphan Asylum, at Bagshot, is appealing for extended support from the charitable public. Over 1,400 children have benefited through this institution, which was established in memory of the Prince Consort.

Donations to the "Corney Grain Memorial Fund" have now reached £725, of which sum £600 has been handed to the governors of the Children's Hospital in Great Ormond Street to endow a cot in memory of Corney Grain.

Paris is inaugurating a scheme this winter to provide the poor and destitute in the streets with shelter and warmth. Awnings and huge braziers are to be erected throughout the city for this purpose, the well-to-do quarters alone being excepted.

The annual meeting of subscribers to the Guest Hospital, Dudley, has been held this month. The total amount received for general purposes has been £3,166 during the period under review. It is gratifying to notice a good increase in the workmen's contributions.

On the whole the report of the Dublin Orthopœdic Hospital is of an encouraging nature. At this institution a ward, especially devoted to the treatment of hip-joint disease in children, has been provided for by the liberality of the late Mrs. Hone, of St. Dolough's Park.

The committee of the Reigate and Redhill Cottage Hospital inform us that the income of the hospital during the past year was not sufficient to meet the outgoings, and that in consequence they have been again compelled to sell out a portion of the invested capital to meet the liabilities.

St. Peter's Hospital for Stone, Henrietta Street, Covent Garden, is doing a good work among sufferers from this distressing disease. Out of 455 patients who passed through the wards last year 13 only succumbed to its evil effects. The patients subscribed £2,121 during the year as a testimony of their gratitude.

Each year the St. Giles' Christian Mission has given a Christmas dinner to several hundred poor families, who, but for its aid, would be quite unprovided for on that day. The funds of this society are exhausted, and contributions addressed to the Secretary, 4, Ampton Street, Regent Square, W.C., are earnestly requested.

The committee of University College Hospital are laying before the benevolent public a few facts showing the widespread usefulness of this charity and its present deplorable financial state. During the past year 3,035 in-patients and 43,767 out-patients have received benefit from this institution, the debt on which at the present critical time exceeds £11,000.

At the annual meeting of the Glasgow Maternity Hospital the chairman expressed his regret that the claims of this valuable institution had been overlooked, and that it did not receive a proper share of the legacies to the charities of the city. The directors are considering ways and means for a further extension of the building for the better accommodation of the patients and students.

The convalescent home connected with the London Medical Mission has been doing a good work this year; it is even projected to enlarge these premises, to admit of further accommodation for patients. Holiday House, in Kent, another offspring of this institution, has received over 260 children and patients during last summer for a fortnight's sojourn in the country. The society is supported by voluntary contributions.

Over one hundred children have benefited through the convalescent home at Weston-super-Mare, an adjunct of the Bristol Children's Hospital. The home is delightfully situated close to the beach, which offers great advantages as a playground for the small inmates. At the present time a debt of £600 exists on the accounts of the institution which the committee are very anxious to have cleared off, as at present interest has to be paid upon it.

### BOOKS RECEIVED.

SWAN SONNENSCHIEN AND CO.

"Text-book of the Embryology of Invertebrates." By Dr. E. Korschelt and Dr. K. Heider, translated from the German by Edward L. Mark and W. McM. Woodworth.

"Public Health in European Capitals." By Thos. Morrison Legge, M.A., M.D.

"Moral Pathology." By A. E Giles, M.D.

H. K. LEWIS.

"An Essay on Malaria and its Consequences." By Robert Lindsay, M.B.

"Treatment of Pulmonary Consumption." By Vincent Dormer Harris, M.D., and Edwin Clifford Beale, M.A., M.D.

BLACKIE AND SON.

"Elementary Inorganic Chemistry." By A. Humboldt Seaton.

"Food and Its Functions." By James Knight, M.A.

OLIVER AND BOYD.

"Notes on Surgery for Nurses." By Joseph Bell, M.D. (Fourth edition).

E. W. ALLEN.

"Perils of Premature Burial." By Dr. Alexander Wilder.

SAMPSON LOW AND MARSTON.

"A Manual of Obstetric Nursing." By Marian Humfrey. (Volume II.).

SCIENTIFIC PRESS.

"Nursing: Its Theory and Practice." By Percy G. Lewis, M.D. (Second edition)

WERTHEIMER, LEA, AND CO.

"Penological and Preventive Principles." By William Sallack, secretary of the Howard Association. (Second and enlarged edition).

FISHER UNWIN.

"Criminal Sociology." By Enrico Ferri, Professor of Criminal Law. (Criminology, Series II.).

KEGAN PAUL AND TRENCH.

"Modern Medicine and Homœopathy." Two addresses by John B. Roberts, A.M., M.D.

WHITTAKER AND CO.

"The Chemists' Compendium." Compiled by C. J. S. Thompson.

JAMES CLARKE AND CO.

"Helps to Health and Beauty." By a Pharmaceutical Chemist.

THE ROXBURGHE PRESS.

"Lying-in." By Marian Humfrey.

J. AND A. CHURCHILL.

"Reasons Why the Office of Coroner Should be Held by a Member of the Medical Profession." By Frederick W. Lowndes.

"Practical Ambulance Tablets." By Sydney Partridge.

ISBISTER AND CO.

"The Tender Mercies of the Good." By Christabel Coleridge.

"The Men of the Moss Hags." By S. R. Crockett.

LEDGER, SMITH, AND CO.

"Appendix to Medical Digest, from 1891 to 1895." By Richard Neall, M.D.

LONGMANS, GREEN, AND CO.

"Hygiene." By J. Lane Notter, M.A., M.D., and R. H. Firth, F.R.C.S.

"Pioneer Work in Opening the Medical Profession to Women." By Dr. Elizabeth Blackwell.

METHUEN AND CO.

"The Vaccination Question." By A. W. Hutton, M.A.

BIGGS AND CO.

"Sewerage and Sewage Disposal of a Small Town." By E. B. Savage, A.M.Inst.C.E.

A. AND C. BLACK.

"A Plea for a Simpler Life." By George S. Keith.

CASSELL AND CO.

"The Herschels and Modern Astronomy." By Clerke.

DIGBY, LONG AND CO.

"Perfect Womanhood." By Frederick James Grant.

**Periodicals and Pamphlets.**—Fiftieth Annual Report of the Sydney Hospital, Edinburgh Medical Missionary Society Quarterly Paper, Monthly Homœopathic Review, Medical Review, Hermes, Birmingham Medical Review, Leeds Hospital Magazine, Toilers of the Deep, Cassell's Family Magazine, The Family Circle, Cider, American Bookmaker, Electrician, Night, Descriptive Sketches of Warneford Hospital, British and Colonial Druggist, Christian World, Electrical Review, Industries and Iron, The Sun, Nursing Record, Nurses' Journal, Medical Press, The Local Government Journal, New York Medical Journal, Medical Week, Sanitary Journal, Charity Organisation Review, Clinical Sketches, Essays by Lady Cook on Social Topics, County Borough of Kingston-upon-Hull: Annual Report of Medical Officer of Health, Service for the King, British Phrenological Year Book, International Medical Magazine, Cambridge Review, Nursing Notes, Nursing World, Monthly Register of Philadelphia Society for Organising Charity, National Review, Public Health, Animals' Guardian, Science, Provincial Medical Journal, Christmas Angel, Galen, Gale Medical Journal, The Princess, Clinical Journal, Medical Bulletin. English Illustrated Magazine, The Chef, Friendly Greetings, Sanitary Record, London, Literary Digest, Revue Générale des Sciences, Popular Science, Review of the Churches, Cornhill Magazine, Westminster Review, Blackwood's Magazine, The Humanitarian, The Quiver, The Verulam Review, Charlotte Medical Journal, Indian Medico-Chirurgical Review, Invention, pures et Appliquées, Review of Reviews, The Sunday at Home, Leisure Hour, Girl's Own Paper, Boy's Own Paper, Building World, The Tri-State Medical Journal, Guy's Hospital Gazette, The Therapist, Pears' Pictorial, Friendly Greetings, The Cottager and Artisan, Parish Councillor, London, Health, The Practitioner,

### THE STUDENT OF MEDICINE.

### APPOINTMENTS.

W. D. Buncombe, L.R.C.P.Lond., M.R.C.S., appointed Medical Superintendent of the City of London Union Infirmary. R. D. Cameron, L.R.C.P., L.R.C.S.Edin., appointed Medical Officer for the Wallingpen District of the Howden Union. G. Carre, M.D., appointed Non-Resident House Surgeon to the Royal Ear Hospital, Frith Street, Soho. W. C. G. Collins, M.R.C.S., L.R.C.P., appointed Medical Officer for the Aylesford District of the Malling Union. W. H. Cooke, M.D.Brux., M.R.C.S., L.R.C.P., L.S.A., appointed Resident Medical Officer to the Royal United Hospital, Bath.

### VACANCIES.

The following vacancies are announced this week, the amount of salary in each case being given after the name of the institution:—

### Resident Medical Officers.

Victoria Hospital for Sick Children, Queen's Road, Chelsea, S.W.—House Surgeon to the In-Patients; appointment for twelve months; must be F. or M.R.C.S.Eng. Honorarium £50 per annum, with board and lodging in the hospital. Also House Physician to the In-Patients; appointment for eight months. Honorarium at the rate of £50 per annum, with board and lodging in the hospital. Applications to the Secretary by January 11th.

North London Hospital for Consumption, Hampstead, N.W.—Resident Medical Officer; doubly qualified. Honorarium £40 per annum, with board, lodging, &c., in the hospital. Appointment for one year (subject to three months' notice on either side), but the elected candidate will be eligible for re-election. Applications to W. G. Farrance Bosworth, Secretary, 41, Fitzroy Square, W., by January 6th.

### Non-Resident Medical Officers.

Central London Throat, Nose, and Ear Hospital, Gray's Inn Road, W.C.—Assistant Registrars. Applications to Richard Kershaw, Secretary, by January 11th.

County Borough of Wigan.—Medical Officer of Health and Medical Superintendent of the Sanatorium. Salary £225 per annum, payable monthly; must reside within the borough. Applications, endorsed "Medical Officer," to J. J. Charnock, Town Clerk, Municipal Buildings, Wigan, by January 6th.

Dental Hospital for London, Leicester Square, W.C.—Assistant Dental Surgeon; must be L.D.S. Applications to J. Francis Pink, Secretary, by January 6th.

Dental Hospital for London and London School of Dental Surgery, Leicester Square, W.C. — Demonstrator. Honorarium £50 per annum. Applications to J. Francis Pink, Secretary, by January 6th.

Samaritan Free Hospital for Women and Children, Marylebone Road, N.W.—Surgeon to the Out-patient Department. Applications to the Secretary, George Scudamore, by January 7th.

### UNIVERSITIES AND COLLEGES.

### UNIVERSITY OF CAMBRIDGE,

#### FIRST M.B. EXAMINATION.

PART I. CHEMISTRY AND PHYSICS.—Bate, King's; Bentley, Joh.; Brown, Emm.; Buttar, B.A., Pemb.; Carey, Emm.; Cave-Moyles, Gonv. and Cai.; Child, Pemb.; Clarke, A. J., Emm.; Claxton, King's; Dixon, G., B.A., Trin.; Elliston, H. Aye.; Fehrsen, Gonv. and Cai.; Fox, W. S., Trin.; Gabb, Down; Geoghegan, Gonv. and Cai.; Gould, Trin.; Hardie, Queens'; Heap, Sid. Suss.; Hedley, J. P., King's; Heffer, Sid. Suss.; Hindle, Corp. Chr.; Holroyde, Trin.; Hudson, A. C., Trin.; Ingram, Trin.: Ingram, A. C., Joh.; Kempthorne, Joh.; Knight, Emm.; Laycock, Joh.; Lee, W. E., Trin.; Loveday, Gonv. and Cai.; McDonnell, Joh.; Naish, B.A., Emm.; Nothwanger, Joh.; Phillpotts, Christ's; Richmond, B.A., Cla; Roberts, J. H., B.A., Joh.; Sedgwick, Sid. Suss.; Sephton, Gonv. and Cai.; Shipman, Trin.; Shipway, Christ's; Simey, B.A., King's; Smedley, Pemb.; Spearman, Gonv. and Cai.; Stiff, Gonv. and Cai.; Stockwell, King's; Taylor, E. J. D., Gonv. and Cai.; Taylor, non-coll.; Whale, Jes.; Wilcock, Gonv. and Cai.; Wilson, G. R., Trin.; Wisdom, Emm.; Woods, Sid. Suss.; Worthington, Trin.

PART II. ELEMENTARY BIOLOGY.—Ball, Emm.; Browne, H. S. D., Trin.; Burrows, Emm.; Carey, Emm.; Carroll, Trin.; Claxton, King's; Cooke, J. G., B.A., Sid. Suss.; Cooper, J. G., Trin.; Deneke, Gonv. and Cai.; Dickinson, W. H., Trin.; Dixon, G., B.A., Trin.; Enthoven, B.A., Gonv. and Cai.; Gabb, Down.; Hawkins, Gonv. and Cai.; Horsley, Gonv. and Cai.; Lawson, G. C., Trin.; Laycock, Joh.; Lushington, Jes.; McDonnell, Joh.; Manser, Pet.; Marcy, G., Trin.; Masterman, H. W., Christ's; Miller, C. H., Trin.; Newton, Pemb.; Parez, Emm.; Perkins, H., Aye; Rees, Down.; Roberts, J. H., B.A., Joh.; Roberts, N. O., Christ's; Ross, E. A., Trin.; Sanders, B.A., Christ's; Sanger, F., Joh.; Scott, C. A. St. J., Trin.; Sedgwick, Sid. Suss.; Skrimshire, Jes.; Spearman, Gonv. and Cai.; Stiebel, Trin. H.; Stiff, Gonv. and Cai.; Stokes, W. H., Pemb.; Stuttaford, H., Selw.; Taylor, E. J. D., Gonv. and Cai.; Wales, Down.; Ward, W. D., Joh.; Wilgress, H. Selw.; Wilkinson, B.A., Pemb.; Woods, Sid. Suss.

#### SECOND M.B. EXAMINATION.

PART I. PHARMACEUTICAL CHEMISTRY.—Ainsworth, B.A., Pemb.; Almond, Emm.; Ascherson, non-coll.; Atkinson, Gonv. and Cai.; Attlee, Joh.; Bainbridge, Trin.; Barnicot, Pemb.; Bell, R. L., B.A., Pet.; Bemrose, Joh.; Bigg, Gonv. and Cai.; Boulton, Trin.; Bousfield, Pemb.; Breddon, Trin. H.; Burnand, Jes.; Carey, Gonv. and Cai.; Crimp, Gonv. and Cai.; Ede, King's; George, Gonv. and Cai.; Gibson, Jes.; Hadfield, Trin.; Hall, D. G., Emm.; Holmes, Gonv. and Cai.; Howard, C. R. Pemb.; Hulbert, H. L. P., B.A., Trin.; James, W. M., B.A., Christ's; Knobel, Trin.; Langton, J. M. E., Trin.; Larner, M.A., Pemb.; Leech, Christ's; Levy, Joh.; Malim, Christ's; Mellor, King's; Mercer, Cla.; Morgan, Joh.; Mummery, Gonv. and Cai.; Orme, Gonv. and Cai.; Percival, Joh.; Pitkin, H., Aye; Roper, B.A., non-coll.; Saunders, C. G., Trin.; Scott, Christ's; Smith, T. D., Jes.; Spanton, Trin.; Stirling-Hamilton, Jes.; Talbot, E., B.A., Trin.; Taylor, C. E., Joh.; Telford, Gonv. and Cai.; Ticehurst, B.A., Cla.; Truman, Trin. H.; Upward, Christ's; Urwick, R. H., Trin.; Weatherhead, Joh.; Whitmore, Gonv. and Cai.; Williams, R. F., Gonv. and Cai.; Woods, W. H. O., H. Selw.; Wright, Christ's.

PART II. HUMAN ANATOMY AND PHYSIOLOGY.—Bates, T. W., M.A., Queens'; Bolland, B.A., Emm.; Brincker, B.A., Joh.; Clarke, H. N., B.A., Gonv. and Cai.; Cox, B.A., Gonv. and Cai.; Curme, B.A., Gonv. and Cai.; Darby, B.A., Trin.; Ellis, B.A., Gonv. and Cai.; Glasier, B.A., Emm.; Glynn, B.A., Cla.; Graham, J. C. W., B.A., Trin.; Harman, Joh.; Harmer, B.A., King's; Hay, B.A., Gonv. and Cai.; Hedley, E. W., B.A., King's; Horne, B.A., Trin.; Hunt, E. R., B.A., Trin.; Keeling, B.A., Gonv. and Cai.; Killick, Down; Lea, J., B.A., Gonv. and Cai.; Le Fleming, E. K., B.A., Cla.; Maxwell, Trin.; Mayo, Cla.; Nicholls, Joh.; Parker, Emm.; Percival, Joh.; Priddle, Gonv. and Cai.; Reid, B.A., Joh.; Rose, B.A., Joh.; Rowland, B.A., H. Selw.; St. Leger, B.A., Gonv. and Cai.; Scaping, B.A., Cla.; Sewell, B.A., Pemb.; Slade, B.A., Trin.; Taylor, J. G., King's; Walker, A. N., B.A., Queens'; Wilkin, B.A., Pemb.; Wilson, A. G., B.A., Gonv. and Cai.; Wilson, E. A., B.A., Gonv. and Cai.

#### THIRD M.B. EXAMINATION, MICHAELMAS TERM, 1895.

PART I. SURGERY AND MIDWIFERY.—Allen, J. L., B.A., King's; Appleyard, B.A., Emm.; Auden, B.A., Christ's; Barker, E. M., B.A., Emm.; Barton, B.A., Joh.; Black, B.A., Trin. H.; Blatchford, B.A., Sid. Suss.; Edwards, C. D., B.A., Joh.; Falkener, L., M.A., King's; Garner, W. L., B.A., Emm.; Harrison, L. K., B.A., Gonv. and Cai.; Haward, B.A., Sid. Suss.; Holmes, H., B.A., Joh.; Howitt, B.A., Gonv. and Cai.; Hutchinson, M.A., Trin. H.; Jackson, T. L., B.A., Joh.; Mackenzie, B.A., Gonv. and Cai.; Maturin, B.A., Gonv. and Cai.; Maxwell, B.A., Corp. Chr.; Milsome, H. B., B.A., Trin.; Moritz, B.A., Gonv. and Cai.; Pendlebury, B.A., Pemb.; Penny, M.A., Pet.; Rawling, B.A., Gonv. and Cai.; Richards, W. G., B.A., Christ's; Robinson, B.A., Gonv. and Cai.; Roper, B.A., Christ's; Salt, B.A., Emm.; Selby, B.A., Down.; Somers, C. D., B.A., Pemb.; Stawell, B.A., Trin. H.; Stead, B.A., Gonv. and Cai.; Tod, H. F., B.A., Trin.; Tyson, B.A., Gonv. and Cai.; Waithman, M.A., Magd.; Ward, A. B., B.A., H. Selw.; Wedd, B.A., Down.; Wills, B.A., Gonv. and Cai.

### PAMPHLETS ON NURSING.

No. 1, price 1½d. per copy, or 1s. per dozen.

**THE ADVANTAGES AND PRIVILEGES OF A TRAINED DISTRICT NURSE, AND THE DUTIES OF THE DISTRICT TOWARDS HER.**

By HENRY C. BURDETT.

No. 2, price 1½d. per copy, or 1s. per dozen.

**A GREAT MOVEMENT—THE NURSES' CO-OPERATION.**

By Mrs. FURLEY SMITH.

London: THE SCIENTIFIC PRESS (LIMITED), 428, STRAND, W.C.

### "THE HOSPITAL"

AN INDEPENDENT JOURNAL OF

**The Medical Sciences**

AND

**Hospital Administration,**

WITH WHICH IS ISSUED WEEKLY

A SPECIAL

### SUPPLEMENT FOR NURSES.

PUBLISHED EVERY SATURDAY.

PRICE TWOPENCE.

Matrons, Sisters, or Nurses will find in THE HOSPITAL a chronicle of the latest Nursing News, Practical Articles of the highest value to those who wish to keep themselves abreast with the times, and brightly written articles from Nurses all over the world.

SUBSCRIPTIONS CAN COMMENCE AT ANY TIME.

### TERMS OF SUBSCRIPTION.

WEEKLY ISSUE.

| | Great Britain. | Foreign and Colonial. |
|---|---|---|
| For One Year ... ... | 10s. 6d. ... | 15s. 2d. |
| For Six Months ... | 5s. 3d. ... | 7s. 7d. |
| For Three Months... | 2s. 8d. ... | 3s. 10d. |

MONTHLY PARTS.

| | Post Free to any Part of the World. |
|---|---|
| For One Year ... ... | 8s. 6d. |
| For Six Months ... ... | 4s. 3d. |
| For Three Months ... ... | 2s. 2d. |

Postal Orders and Cheques should be made payable to "The Scientific Press, Limited."

N.B.—In order to prevent delay, all business communications should be addressed to "THE MANAGER" only, and not to any person individually.

CHANGE OF ADDRESS.

In event of change of address, notice should be forwarded to the Publishers by the Saturday previous to date of publication, and the old address should also be given.

"THE HOSPITAL" can be obtained of all Booksellers and Newsagents in the United Kingdom, and at all the Railway Stalls, of Messrs. W. H. Smith and Son; and also from the following Agents in the Colonies and Abroad:—

PARIS: Librairie Galignani, 224, Rue de Rivoli.
BERLIN: Messrs. A. Asher and Co.
LEIPSIG: Mr. F. A. Brockhaus.
NEW YORK: The International News Co.
MONTREAL: The Montreal News Co.
TORONTO: The Toronto News Co.
MELBOURNE, SYDNEY, ADELAIDE, and NEW ZEALAND: Messrs. Gordon and Gotch.
INDIA: At all the Railway Bookstalls and Agencies of Messrs. A. H. Wheeler and Co.
SOUTH AFRICA: Messrs. Juta, Cape Town.

PUBLISHING OFFICES: 428, STRAND, LONDON, W.C.

### THE NURSES' BOOKSHELF.

Crown 8vo., 500 pp., Second Edition, 7 Plates 18 Illustrations, Charts, &c , price 7s. 6d. net.

**NURSING.** By ISABEL ADAMS HAMPTON.

"It is not too much to say that in no single volume yet published has the subject been so scientifically, carefully, and completely treated as it has been by Miss Hampton. . . . . Whether as a teaching manual or as a book of reference the thoroughly practical instruction which it conveys is sure to obtain a wide circulation."—*The Hospital.*

Demy 8vo, 200 pp., cloth extra. Price 3s. 6d.

**SURGICAL WARD-WORK AND NURSING.** A practical Manual of Clinical Instruction for Nurses and Students. This book will be found especially useful to beginners, to whom its simple description of elementary principles and treatment in surgery will render it invaluable. By ALEXANDER MILES, M.D. (Edin.), C.M., F.R.C.S.E. Over 100 Illustrations.

"Will furnish much-needed guidance to the student and the nurse in the early stages of their apprenticeship."—*Times.*

Third Edition. Enlarged and partly rewritten, cloth gilt, crown 8vo, about 200 pp., price 2s. 6d post free.

**HOSPITAL SISTERS AND THEIR DUTIES.** By EVA C. LUCKES, Matron to the London Hospital. The favourable reception accorded to the first two editions of this useful and interesting work, and the regular demand for copies of it, encourage the publishers to place this third edition, greatly improved and enlarged, before the Institutions. The experience and position of its author entitle it to authority, and every Matron, Sister, or Nurse who aspires to become a Sister should read the advice and information contained in its pages.

Demy 8vo, nearly 200 pp., cloth gilt, profusely illustrated with over 70 special cuts. Price 6s. (post free).

**THE ART OF MASSAGE.** By A. CREIGHTON HALE. A complete Text-book of this valuable art. The most practical, simple, and generally useful work published on the subject. With fully illustrated chapters on the Anatomy of the Human Body.

Demy 8vo., cloth extra, price 3s. 6d., post free. Popular Edition, stitched, price 1s. 6d.

**OUTLINES OF INSANITY.** A Popular Treatise on the Salient Features of Insanity. By FRANCIS H. WALMSLEY, M.D., Medical Superintendent of the Darenth Asylum, Metropolitan Asylums Board, Member of Council of Medico-Psychological Association.

A clearly written manual in which the various features of mental disorder are fully expounded. The author's style is interesting, and the reader's attention is riveted throughout.

Crown 8vo., cloth gilt, with many Plates and Illustrations in the text, price 2s. 6d., post free.

**SPINAL CURVATURE AND AWKWARD DEPORTMENT:** Their Causes and Prevention in Children. By Dr. GEORGE MULLER, Professor of Medicine and Orthopædics, Berlin. English Edition, edited and adapted by RICHARD GREENE, F.R.C.P.

This work will be found to be a handy and authoritative manual on the prevention and treatment of spinal curvature and awkward deportment in children.

London: THE SCIENTIFIC PRESS (Limited), 428, STRAND, W.C.

*In the Press. Price 5s.*

### BURDETT'S OFFICIAL NURSING DIRECTORY

A DIRECTORY, NOT A REGISTER.

The Hospital states: "It is well to bear in mind the difference and distinction which exists between a register and a directory. A register implies rights, and suggests that those who are not upon it are in some way or another inferior beings. But a directory is another matter. A directory gives as rights; insertion in it implies no adherence to any particular party or any particular opinion, and in no way interferes with entry on the register of any association, or the list of any institute, to which the individual may belong. All that a directory professes to do is to give information. The 'Medical Register' is not a mere list of qualified medical men; it is admission to the Register, which itself makes him qualified. For years, perhaps even now, 'Churchill's Medical Directory' contained the names of medical men who, so far as public appointments were concerned, were unqualified; it also contains the names of homœopathists, whom four out of every five medical men will not meet; and it certainly contains the names of many for whose moral character we should not like to vouch. But it gives full and accurate information about them all, and for the sake of that information it is constantly consulted both by the public and the doctors."

What Nurses Need.

"At present there is no Register for the nursing profession giving any legal status. Such a thing may come or it may not; but in either case there is no reason why there should not be a Directory, giving such information about the career of each nurse as both the public and the profession want; information, by the by, which no legal Register would be likely to insert. The 'Medical Register' is a bald and meagre thing compared with the 'Medical Directory.' What Burdett's 'Official Nursing Directory' proposes to do is to supply the want of a directory giving information. It will give no rights, it will imply no partisanship, it will give no guarantee except that what it prints is true, and is derived from official sources."

Acceptable to All Nurses.

"This ought to be enough to make it acceptable to the public, and every nurse ought to make sure that, whatever list or register she may be on, her name shall also appear on Burdett's 'Official Directory.'" Applications for forms should be made at once to the Editor, "Burdett's Official Nursing Directory," 428, Strand, W.C.

**The above work will be ready early in the coming year, and those Nurses who have not already sent in their names should at once apply for a form, which can be obtained upon application to the Publishers,**
THE SCIENTIFIC PRESS (Lim.),
428, Strand, London, W.C.

### NURSING BOOKS AT FULL DISCOUNT.

*LIST POST FREE.*

The Theory and Practice of Nursing (Percy Lewis, M.D.), 3/6 for **2/8.**

The Nurse's Dictionary (Honnor Morten), 2/- for **1/6.**

The Nurse's Case Book, 6d. for **3½d.**

